# On the Identity of the Nervous and Electric Fluid

**Published:** 1816-02

**Authors:** T. Purton

**Affiliations:** Member of the Royal College of Surgeons.


*For the London Medical and Physical Journal.*

*On the Identity of the Nervous and Electric Fluid;* by T. Purton, Member of the Royal College of Surgeons.

IN the 192d Number of your Journal for February last, I was much gratified in finding the same hypothesis revived, respecting the identity of the electric and nervous fluid, which I published in your Journal many years ago.* I hope Mr. Smerdon will continue his researches, as he appears to possess that peculiar acumen for investigating intricate subjects, which is so essential to the elicitation of the truth.

*Alcester, Warwickshire;*
*January* 10, 1816.

---

* Vol. IV. p. 334, and Vol. VII. p. 324.

